# Prospective Genotyping of *Mycobacterium tuberculosis* from Fresh Clinical Samples

**DOI:** 10.1371/journal.pone.0109547

**Published:** 2014-10-14

**Authors:** Urška Bidovec-Stojkovič, Katja Seme, Manca Žolnir-Dovč, Philip Supply

**Affiliations:** 1 Laboratory for Mycobacteria, University Clinic of Respiratory and Allergic Diseases, Golnik, Slovenia; 2 University of Ljubljana, Faculty of Medicine, Institute of Microbiology and Immunology, Ljubljana, Slovenia; 3 INSERM U1019, Lille, France; 4 CNRS UMR 8204, Lille, France; 5 University of Lille Nord de France, Lille, France; 6 Institut Pasteur de Lille, Center for Infection and Immunity of Lille, Lille, France; 7 Genoscreen, Lille, France; St. Petersburg Pasteur Institute, Russian Federation

## Abstract

Shorter time-to-result is key for improving molecular-guided epidemiological investigation of tuberculosis (TB) cases. We performed a prospective study to evaluate the use of standardized MIRU-VNTR (mycobacterial interspersed repetitive-unit-variable-number tandem-repeat) typing of *Mycobacterium tuberculosis* directly on 79 fresh clinical samples from 26 TB patients consecutively enrolled over a 17-month period. Overall, complete 24-locus types were obtained for 18 out of the 26 (69.2%) patients and 14 of the 16 grade 3+ and grade 2+ samples (87.5%). The degree of completion of the genotypes obtained significantly correlated with smear microscopy grade both for 26 first samples (*p* = 0.0003) and for 53 follow-up samples (*p* = 0.002). For 20 of the 26 patients for whom complete or even incomplete *M. tuberculosis* isolate genotypes were obtained, typing applied to the clinical samples allowed the same unambiguous conclusions regarding case clustering or uniqueness as those that could have been drawn based on the corresponding cultured isolates. Standard 24 locus MIRU-VNTR typing of *M. tuberculosis* can be applied directly to fresh clinical samples, with typeability depending on the bacterial load in the sample.

## Introduction

Molecular typing of pathogens has become an important tool in clinical microbiology and disease surveillance. This is especially true for contagious diseases such as tuberculosis (TB). It is estimated that in 2011 there were over 8.7 million new TB cases causing 1.4 million deaths [1]. The emergence of multidrug-resistant (MDR) and extensively drug-resistant (XDR) strains of *M. tuberculosis* strains is of global concern [2]. In the European Union alone, the economic burden resulting from TB has been estimated at more than €5 billion [3]. In addition to new vaccines and drugs, effective tools are needed to better trace, control, and prevent TB transmission. Without effective implementation of control and preventive measures, TB outbreaks can rapidly develop even in population groups thought to be at very low risk for TB [4].

As in a number of other countries in the western world, molecular epidemiology has become an important part of TB surveillance in Slovenia. Molecular-guided TB control was adopted years ago, when a nationwide molecular epidemiological study identified important risk factors and new routes of TB transmission [5]. Implementation of a molecular epidemiology program in 1999 coincided with a substantial decrease of TB incidence in Slovenia from 19.1/100,000 inhabitants in 2000 to 6.7/100,000 in 2012. Initially based on IS*6110* restriction fragment length polymorphism (RFLP), molecular surveillance has been performed since 2009 based on standard MIRU-VNTR typing [6]. The use of this faster, PCR-based technique greatly reduces the time required to identify clusters from cultured samples. Although it does not attain the discriminatory power of whole-genome sequencing [7], [8], MIRU-VNTR typing, whether combined with spoligotyping or not, has been shown to have a predictive value similar to that of IS*6110* fingerprinting for tracing TB transmission at the population-based level in several European countries, including Slovenia [9]–[13].

However, one challenge remains unsolved; namely, how to obtain the *M. tuberculosis* genotyping results sufficiently quickly to integrate them into the initial survey of TB transmission while prospective contact tracing is still ongoing. Because of the slow growth of the organism, the usual typing from cultured *M. tuberculosis* is most often too slow for timely use at this early stage, and more rapid methods are needed [13]. Such fast genotyping would be especially helpful in countries like Slovenia, where TB patients are treated in hospitals as long as their microscopy results are positive. Such an approach would be valuable for better identifying epidemiological links while the patient is still in the hospital near the epidemiology department or for more rapidly ruling out potential relapse of TB or laboratory cross-contaminations. Direct genotyping of samples seems to be the ideal objective for timely genotyping results.

Few studies have indicated the potential for direct MIRU-VNTR typing of *M. tuberculosis* from clinical samples [14], [15]. A first study included preselected frozen samples, [14], while a second one included smear positive samples from TB patients in a prison TB hospital in Kyrgyzstan [15]. In our study, we prospectively tested whether fresh clinical samples from a general population with a wide range of bacterial loads - down to 3 to 6 bacilli per 100 fields, such as routinely observed in most clinical laboratories - are suitable for *M. tuberculosis* typing, using standardized 24-locus MIRU-VNTR typing kits and routine conditions also used for typing *M. tuberculosis* from culture. Moreover, we included up to 10 longitudinal samples per patient, in order to test the typeability of clinical samples obtained at different treatment stages, an additional parameter that was not investigated in previous studies.

## Materials and Methods

### Ethics statement

The study was approved by the University Clinic of Respiratory and Allergic Diseases Golnik and was performed in accordance with the Declaration of Helsinki. All participants gave written informed consent according to the guidelines of the University Clinic for Respiratory and Allergic Diseases Golnik ethical review board prior to their inclusion in the present study.

### Study design

In one group, we included initial smear-positive sputum samples obtained from 26 patients, all new TB cases consecutively identified at the University Clinic of Respiratory and Allergic Diseases Golnik between October 2011 and January 2013. Immediately after a positive microscopic result, the physician in charge was asked for a new sample, which was used for complete mycobacterial analysis, including direct genotyping. Samples were collected at the TB Department at the clinic, 1 to 15 days after initiation of the treatment (related to condition of the patient) and transported within 1 hour to the National Reference Laboratory for Mycobacteria in the same building.

In another group, we included the 53 follow-up samples collected in total from all the patients mentioned above. Follow-up samples were preferably taken twice a week until the first negative smear result in order to test whether time since initiation of the treatment could influence the typing results. Follow-up samples were used to determine the significance of sample collected time.

### Processing of clinical samples

All of the samples collected were sputa the most common sample for TB identification and were handled according to the TB laboratory's standard procedures. Sample volumes ranged from 5 to 20 ml. Whole samples were subjected to a decontamination process (performed in aliquots when the volume exceeded 5 ml). Smear microscopy by Auramine was performed on the same day. Based on the number of acid-fast bacilli (AFB) observed by smear microscopy, clinical samples were classified into five groups:>10 bacilli/field (3+), 1–10 bacilli/field (2+), 10–99 bacilli/100 fields (1+), 7–9 bacilli/100 fields (2 AFB), and 3–6 bacilli/100 fields (1 AFB) [12]. For a conventional mycobacterial culture, 500 µl of decontaminated sample was inoculated onto Lowenstein–Jensen (LJ) solid media and in MGIT liquid media, incubated at 37°C and monitored for growth following standard laboratory protocols [16]. Also in contrast to the previous study that used DNA purification on column [11], DNA was extracted from 1–2 ml of decontaminated sputum using the same routine crude extraction protocol after heat inactivation as used and described earlier for *M. tuberculosis* cultures [17], [18], [19].

### Genotyping

PCR amplification of 24 MIRU-VNTR loci was performed with a standardized MIRU-VNTR Typing Kit, triplex version (GenoScreen, Lille, France), as described in the manufacturer's manual. PCR were performed using 96-well plates, each one including 10 samples, one positive (H37Rv or *M. bovis* BCG Pasteur included in the kit) and one negative (water) control subjected to eight triplex PCRs. For each triplex PCR, 2 µl of extracted DNA were added to 8 µl of ready-to-use PCR reaction mixture. The amplicons were analyzed on a four-capillary-based ABI 3130 genetic analyzer (Applied Biosystems, USA), previously calibrated with the MIRU-VNTR Calibration Kit (GenoScreen, Lille, France). PCR fragment sizing and assignment of the alleles of the 24 loci were done using GeneMapper software v. 4.0 (Applied Biosystems, USA). The reproducibility and accuracy of sizing were checked by analyzing the PCR fragments amplified from the *M. tuberculosis* H37Rv (or BCG, see above) positive control. Analysis of typing results was performed with BioNumerics program ver. 5.1 (Applied Maths, Belgium). When full 24-locus MIRU-VNTR results were not obtained in the first round, PCR and fragment analysis were repeated. The second result was final for analysis. The MIRU-VNTR genotype result obtained from each clinical sample was compared with the MIRU-VNTR genotype obtained from the corresponding cultured *M. tuberculosis* isolate.

### Statistical evaluation

The distribution of data was determined using the D'Agostino–Pearson omnibus normality test. The Spearman rank correlation test was used to analyze the degree of linear association between the degrees of completion of MIRU-VNTR genotypes obtained and smear microscopy grade for first and follow-up samples. Statistical analyses were performed using GraphPad Prism 5 software (San Diego, CA, USA) and probability values of *p*<0.05 were accepted as significant.

## Results

Out of 26 patients' first samples included in the first part of the study, 13/26 (50%) were highly positive (3+); the rest had a lower bacterial load, including 3/26 with grade 2+, 5/26 with grade 1+, 2/26 with grade 2AFB, and 3/26 with grade 1AFB (Table S1).

More than half of the first samples, 16/26 (61.4%), were fully typed and successful typing was associated with a higher bacterial load because 14 of these 16 samples (87.5%) that were typed completely consisted of grade 3+ and 2+ samples (Table S1).

**Figure 1:**
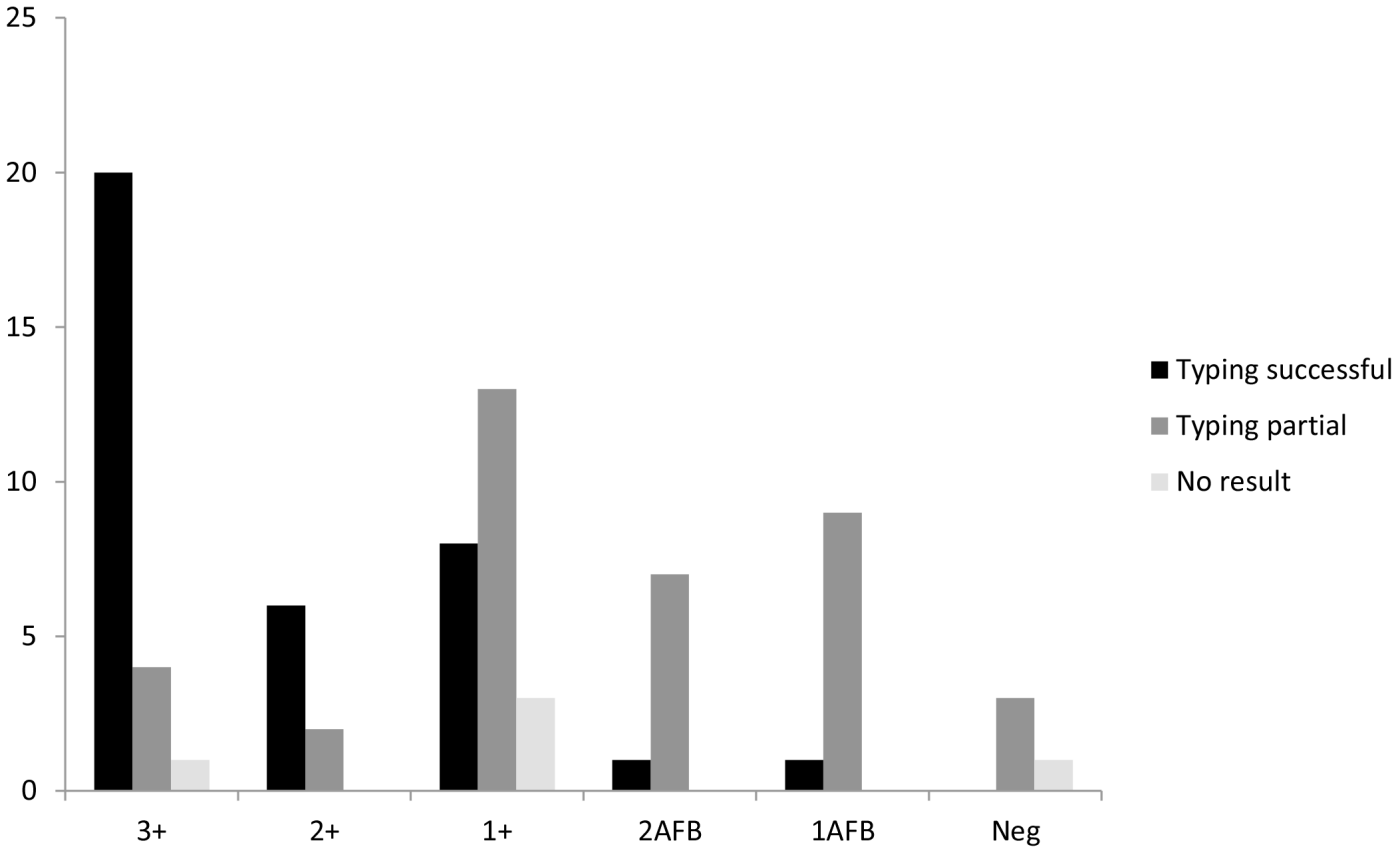
Success of MIRU-VNTR typing in correlation with smear microscopy. MIRU-VNTR typing results of all 79 samples taken from 26 TB patients in correlation with smear microscopy result. Smear microscopy grades are coded as follows: 3+ (>10 bacilli/field); 2+ (1–10 bacilli/field); 1+ (10–99 bacilli/100 fields); 2AFB (7–9 bacilli/100 fields), and 1 AFB (3–6 bacilli/100 fields).

We then extended our analysis to the 53 follow-up samples collected from the same 26 patients and analyzed the results based on all 79 clinical samples (Table 1 and Figure 1). Out of 79 samples, 36 (45.6%) were typed completely, 38 (48.1%) partially (with 1 to 23 loci amplified out of the 24 targets), and five (6.3%) were considered completely untypeable (no locus amplified) for *M. tuberculosis*.

**Table 1:** MIRU-VNTR typing results of all 79 samples, taken from 26 patients, depending on initiation of TB treatment and result of smear microscopy.

START OF TB TREATMENT (D = DAY/WEEK)		PATIENT (NAME CODE)	MICROSCOPY	580	2996	802	960	1644	3192	424	577	2165	2401	3690	4156	2163b	1955	4052	154	2531	4348	2059	2687	3007	2347	2461	3171
0D	1 WEEK	GAL	3+	2	4	2	3	3	3	2	4	3	2	1	2	3	2	5	2	6	2	2	1	3	4	2	3
0D		OKA	1AFB	3	5	5	3	3	2	3	4	4	2	3	2	na	1	4	2	5	2	2	1	2	4	2	3
1D		KSA	3+	2	5	4	2	3	3	2	4	3	2	3	2	3	2	5	2	5	2	2	1	3	4	2	3
1D		HMI	3+	2	5	2	4	3	3	2	3	3	4	4	3	4	3	6	2	5	2	2	1	3	4	2	3
1D		OKA	1+	3	5	5	na	na	2	3	4	4	2	3	na	na	1	na	2	5	2	2	1	2	4	2	3
2D		RKA	2AFB	na	na	na	na	na	na	na	na	na	na	1	na	na	na	na	na	na	na	na	na	na	na	na	na
3D		KSA	3+	2	5	4	2	3	3	2	4	3	2	3	2	3	2	5	2	5	2	2	1	3	4	2	3
3D		HHA	2AFB	2	4	4	na	na	na	2	3	3	4	3	na	na	6	4	2	5	1	2	1	3	4	2	3
4D		IAS	3+	2	5	2	4	3	3	2	3	3	4	4	3	4	3	6	2	5	2	2	1	3	4	2	3
4D		RFR	3+	2	5	3	3	1	3	2	4	3	2	7	2	3	2	5	2	5	2	2	1	3	4	2	3
4D		BHA	3+	2	5	3	na	na	na	2	3	3	4	3	na	3	3	5	2	5	2	2	1	3	4	2	3
5D		HDA	3+	2	5	2	4	3	3	2	3	3	4	4	3	4	3	6	2	5	2	2	1	3	4	2	3
7D		MRU	1+	3	5	5	3	3	2	3	4	3	2	3	2	4	1	4	2	5	2	2	1	2	4	2	3
8D	2 WEEKS	KMA	3+	2	5	4	3	1	3	2	4	2	2	5	2	2	2	5	2	5	2	2	1	3	4	3	2
8D		MMI	3+	2	5	1	3	2	3	2	3	3	4	4	3	6	3	7	2	5	1	2	1	3	4	2	3
8D		LMU	2+	2	5	2	5	3	3	2	3	3	4	3	3	4	3	6	2	5	2	2	1	3	4	2	3
8D		HMI	1+	na	5	na	na	na	na	na	3	3	na	na	na	na	3	na	na	na	na	2	na	na	4	na	na
10D		KSA	3+	2	5	4	2	3	3	2	4	3	2	3	2	3	2	5	2	5	2	2	1	3	4	2	3
10D		HHA	1+	2	4	4	na	na	na	2	3	3	4	3	na	6	6	4	na	na	na	na	na	na	4	2	3
11D		IAS	3+	2	5	2	4	3	3	2	3	3	4	4	3	4	3	6	2	5	2	2	1	3	na	na	na
11D		ČIV	2+	2	5	2	4	3	3	2	3	3	4	4	3	4	3	6	2	5	2	2	1	3	4	2	3
11D		RFR	1+	na	na	na	na	na	na	na	na	na	na	na	na	na	na	na	na	na	na	na	na	na	na	na	na
12D		MRU	2AFB	3	5	5	3	3	2	3	4	3	2	3	2	4	1	4	2	5	2	2	1	2	4	2	3
12D		BHA	3+	2	5	3	3	3	3	2	3	3	4	3	3	3	3	5	2	5	2	2	1	3	4	2	3
13D		BFI	3+	2	5	3	3	3	3	2	4	2	2	3	2	3	2	3	2	6	2	2	1	3	4	2	3
13D		LMU	3+	2	5	2	5	3	3	2	3	3	4	3	3	4	3	6	2	5	2	2	1	3	4	2	3
13D		HKA	1+	2	na	na	na	na	na	na	na	na	na	na	na	na	na	na	na	na	na	na	na	na	na	na	na
14D		KSA	3+	2	5	4	2	3	3	2	4	3	2	3	2	3	2	5	2	5	2	2	1	3	4	2	3
14D		KVI	1+	2	5	3	4	3	2	1	2	2	1	2	na	1	3	6	1	6	2	2	1	3	4	2	3
14D		SJE	1+	2	5	2	3	2	3	2	3	3	2	3	2	4	2	4	2	5	2	2	1	3	4	2	3
14D		MMI	2AFB	2	5	1	na	na	na	2	3	3	na	na	na	na	3	na	2	5	2	2	na	na	4	2	3
14D		BHA	3+	2	5	3	3	3	3	2	3	3	4	3	3	3	3	5	2	5	2	2	1	3	4	2	3
15D	3 WEEKS	KVI	1+	2	5	3	4	3	2	1	2	2	1	2	2	1	3	6	1	6	2	2	1	3	4	2	3
15D		TVI	3+	4	5	6	3	1	2	3	4	3	2	3	na	3	2	5	2	5	2	2	1	3	4	2	3
15D		RFR	1+	2	5	3	3	1	3	2	4	3	2	7	2	3	2	5	2	5	2	2	1	3	4	2	3
15D		BMA	3+	2	5	3	5	3	3	2	3	3	4	3	3	4	3	4	2	5	2	2	1	3	4	2	3
16D		JMI	2+	4	5	4	3	3	3	4	3	3	2	3	2	5	1	9	3	5	2	2	1	3	2	2	3
17D		HHA	NEG	2	4	na	na	na	na	2	3	3	na	na	na	na	na	na	na	na	na	na	na	na	na	na	na
17D		HMI	2+	2	5	2	na	na	na	2	3	na	4	na	na	4	3	na	2	na	2	2	1	3	4	2	3
18D		BFI	2+	2	5	3	3	3	3	2	4	2	2	3	2	3	2	3	2	6	2	2	1	3	4	2	3
19D		MRA	3+	1s	5	3	5	3	3	2	3	3	4	3	3	2	3	5	2	5	2	2	1	3	4	2	3
20D		IAS	3+	2	5	2	na	na	na	2	3	na	4	na	na	na	3	na	2	na	2	2	1	3	4	2	3
20D		LMU	1+	2	na	2	na	na	na	na	3	3	na	3	na	4	na	na	2	5	na	na	1	na	4	2	na
20D		KKL	1AFB	2	na	2	na	na	na	na	na	na	na	3	na	na	na	na	na	na	na	na	na	na	4	2	3
21D		KSA	3+	2	5	4	2	3	3	2	4	3	2	3	2	3	2	5	2	5	1	2	1	3	4	2	3
21D		BHA	1+	2	5	3	3	na	3	2	3	3	4	3	na	3	3	5	2	5	2	2	1	3	4	2	3
22D	4 WEEKS (1 MONTH)	HHA	2AFB	2	4	4	4	na	3	2	3	3	4	3	na	na	6	4	2	5	2	2	1	3	4	2	3
22D		HMI	2+	2	5	2	na	na	na	2	3	3	4	na	na	na	3	na	2	5	na	2	1	3	4	2	na
22D		RFR	2AFB	2	5	3	3	1	3	2	4	3	2	na	2	na	2	5	2	5	2	2	1	3	4	2	3
24D		KSA	3+	2	5	4	2	3	3	2	4	3	2	3	2	3	2	5	2	5	2	2	1	3	4	2	3
25D		IAS	3+	2	5	2	4	3	3	2	3	3	4	4	3	4	3	6	2	5	2	2	1	3	4	2	3
25D		BFI	1+	na	na	na	na	na	na	na	na	na	na	na	na	na	na	na	na	na	na	na	na	na	na	na	na
25D		RFR	1AFB	2	5	3	na	na	na	2	4	3	2	na	na	3	2	5	2	5	1	2	1	3	4	2	3
26D		BHA	1+	2	5	na	na	na	na	2	3	3	na	3	na	3	3	na	2	5	2	2	1	na	4	2	na
28D		KSA	1+	2	5	4	2	na	3	2	4	3	2	3	na	3	2	5	2	5	2	2	1	3	4	2	3
28D		BHA	1+	na	na	na	na	na	na	na	3	na	na	na	na	na	na	na	na	na	na	na	na	na	na	na	na
29D	5 WEEKS	KSA	2+	2	5	4	2	3	3	2	4	3	2	3	2	3	2	5	2	5	2	2	1	3	4	2	3
29D		HMI	1AFB	na	na	na	na	na	na	na	3	3	na	na	na	na	na	3	2	na	na	na	na	na	na	na	na
30D		RFR	2+	2	5	3	3	1	3	2	4	3	2	2	2	3	2	5	2	5	2	2	1	3	4	2	3
31D		ČIV	2AFB	2	5	2	na	na	na	2	3	3	4	4	3	4	3	6	2	5	2	2	1	3	4	2	3
32D		HHA	NEG	na	na	na	na	na	na	na	na	na	na	na	na	na	na	na	na	na	na	na	na	na	na	na	na
32D		BFI	1+	na	na	na	na	na	na	na	na	na	na	na	na	na	na	na	na	na	na	na	na	na	na	na	na
33D		BHA	1+	2	5	3	3	3	3	2	3	3	4	3	na	3	3	5	2	5	2	2	1	3	4	2	3
35D		BHA	1+	2	5	3	na	na	na	2	3	3	4	3	3	3	3	na	2	5	2	2	1	3	4	2	3
36D	6 WEEKS	KSA	1+	2	5	4	2	3	3	2	4	3	2	3	2	3	2	5	2	5	2	2	1	3	4	2	2
36D		HHA	1AFB	2	4	na	na	na	na	2	3	3	4	3	na	na	na	na	2	5	1	na	1	3	4	2	3
36D		HMI	3+	na	na	na	na	na	na	na	na	na	na	na	na	na	na	na	na	na	na	na	na	na	na	na	na
37D		RFR	1AFB	2	5	3	3	1	3	2	4	3	2	na	na	3	2	5	2	5	1	2	1	3	4	2	3
38D		KSA	1+	2	5	4	2	3	3	2	4	3	2	3	2	3	2	5	2	5	2	2	1	3	4	2	3
41D		LMU	2AFB	2	5	2	na	na	na	2	na	na	na	na	na	na	3	na	2	5	na	na	na	na	na	na	na
42D		LFR	1+	4	5	4	3	na	3	4	3	3	2	3	na	5	1	na	3	5	na	2	1	3	2	2	3
43D	7 WEEKS AND MORE	HHA	1AFB	2	4	4	4	na	na	2	3	3	4	3	na	na	6	4	2	5	2	2	1	3	4	2	3
43D		BHA	1+	2	5	3	3	3	3	2	3	3	4	3	3	3	3	5	2	5	2	2	1	3	4	2	3
47D		BHA	1+	2	5	3	3	3	3	2	3	3	4	3	3	3	3	5	2	5	2	2	1	3	4	2	3
50D		HMI	NEG	2	5	2	na	na	na	na	na	na	4	na	na	na	na	na	2	5	na	na	na	na	na	na	na
51D		DMA	1AFB	1	5	3	na	na	na	2	3	3	5	4	na	na	3	6	2	na	na	2	1	3	4	2	3
53D		IAS	NEG	na	na	2	na	na	na	2	na	na	na	na	na	na	na	na	na	na	na	na	na	na	na	na	na
70D		RFR	1AFB	2	5	3	3	1	3	2	4	3	2	na	na	3	2	5	2	5	1	2	1	3	4	2	3
83D		KSA	1AFB	2	5	4	2	3	3	2	4	3	2	3	2	3	2	5	2	5	2	2	1	3	4	2	3
			No. of mistakes per loci	10	12	13	30	35	30	13	11	14	17	21	38	26	15	24	13	17	20	17	17	19	14	15	18
			%	7.9	15.2	16.4	38	44.3	38	16.4	14	17.7	21.5	26.6	48.1	33	19	30.3	16.4	21.5	25.3	21.5	21.5	24	17.7	19	22.8

Missing alleles are coded as *na* (i.e., non amplified).

When the results of the clinical isolates were compiled at the patient level, *M. tuberculosis* was completely typed directly for 18 out of 26 (69.2%) patients, and in another eight (30.8%) patients 24-locus MIRU-VNTR genotype results were partial, with a minimum of 16 loci amplified.

**Figure 2:**
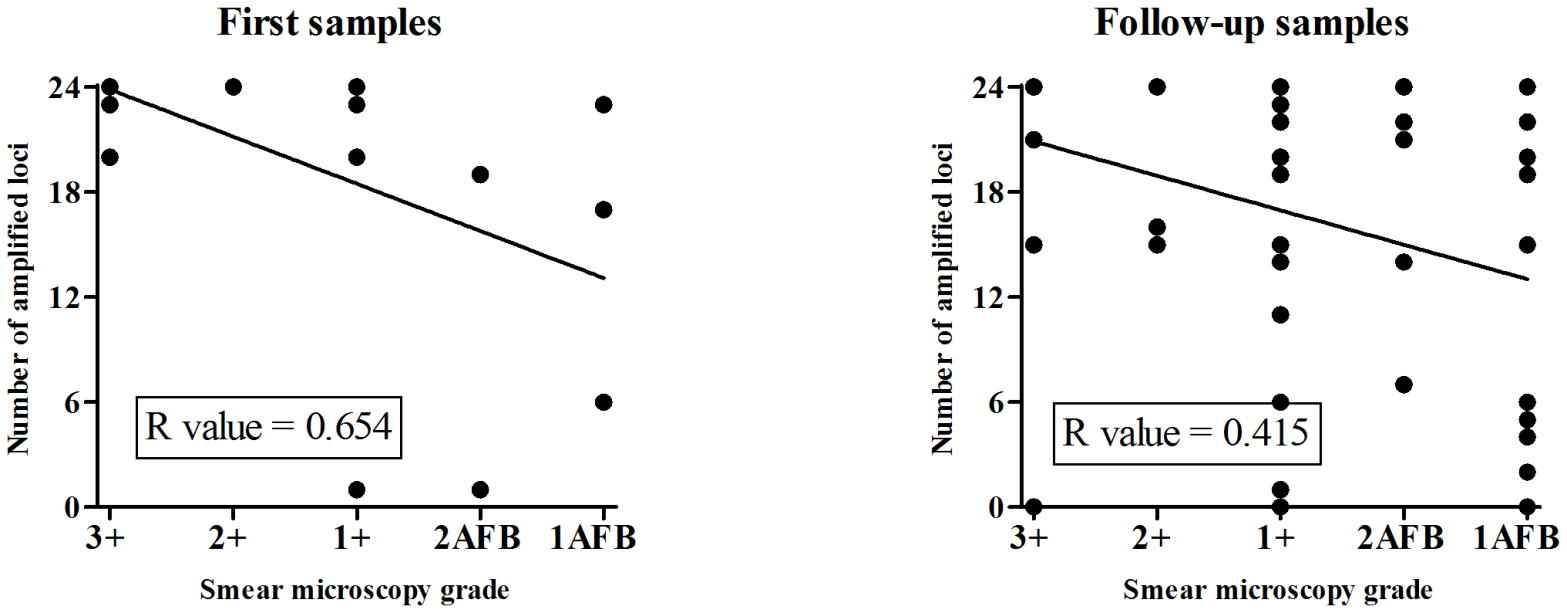
The number of amplified loci in correlation with smear microscopy. The number of amplified loci in correlation with smear microscopy grades is shown by dots for first (A) and follow-up (B) samples. The calculated curves and coefficients (*R*) of linear correlation are also indicated for both sample sets. Smear microscopy grades are coded as follows: 3+ (>10 bacilli/field); 2+ (1–10 bacilli/field); 1+ (10–99 bacilli/100 fields); 2AFB (7–9 bacilli/100 fields), and 1 AFB (3–6 bacilli/100 fields).

There was a significant positive correlation between the number of loci successfully amplified and the bacterial load, both for the first (*p* = 0.0003) and the follow-up samples (*p* = 0.002). That is, the number of alleles obtained was higher when the bacterial load was higher (Table 1, Figure 2, Table S2). The same sensitivity to bacterial load was also noticed at the individual locus level because the most frequently non-amplified locus overall, 4156, was missing in 16% of 3+, 25% of 2+, 62.5% of 1+, 62.5% of 2AFB, and 80% of 1AFB samples. This locus was also the most frequent single locus missing when the 23 other loci were successfully amplified (three out of four cases; in the remaining case, locus 2163b was missing). In terms of non-amplification frequencies, this locus was followed by loci 1644, 960, and 3192. As could be expected, we generally observed lower proportions of complete *M. tuberculosis* types among samples that were collected only after the first 3 weeks of treatment, probably reflecting their often lower bacterial loads (Table 1).

When we compared our data from direct genotyping on clinical samples with the MIRU-VNTR genotypes obtained by analyzing the corresponding cultured isolates, the results were fully identical for all of the samples (data not shown).

We then analyzed the distribution of unique and clustered cases based on both full or partial genotypes among our prospectively collected samples, after excluding two patients for which all samples available displayed genotypes with fewer than 5 alleles (i.e. we retained genotypes minimally matching a 5-locus format such as the first historical, so-called ETR VNTR typing format) [20]. For incomplete genotypes, cluster analysis was done only on the basis of the markers for which an allele was obtained. Among the 24 TB patients retained, sixteen cases were found to be unique on the basis of complete or most complete isolate genotypes identified. Four other patients were found to be clustered in a same single cluster by complete 24-locus strain genotypes. For these 20 patients, typing applied on clinical samples thus allowed the same unambiguous conclusions regarding case clustering or uniqueness as those that could have been drawn based on the corresponding cultured isolates. The 4 remaining cases were found in two separate clusters, each including a sample with a complete genotype and another sample with an incomplete genotype (with 4 or 18 alleles missing, respectively). For both samples with incomplete genotype based on direct typing, the complete genotypes that were obtained from the corresponding, subsequently obtained isolates after culture were found to be distinct in one case, and in the other case identical to that of the other sample in the cluster considered. Thus, taking into account these cases, our direct typing on clinical samples produced overall results that were as conclusive as those based on subsequent cultures for 22 out of 26 TB cases, although in two cases the result was ambiguous because of the incompleteness of a clustered genotype.

Finally, we compared the frequencies of different non-amplified MIRU-VNTR loci among the 79 samples of this prospective study (mostly found among samples with a low bacterial load; see above) with the allelic diversities of the same loci among 919 isolates from a countrywide population-based study (Table 2). As shown in Table 2, locus 4156, which was the most frequently non-amplified locus from the clinical samples, was among the least diverse loci in the nationwide collection, with only two major alleles representing >95% of the locus variability. Locus 2163b and other loci showed more diverse allelic distributions in the nationwide collection.

**Table 2:** Allelic diversities of 24 MIRU-VNTR loci of Slovenian *M. tuberculosis* isolates (*n* = 919) from a retrospective population-based study and proportion of missing allele per locus in our prospective study from direct sputum samples (*n* = 79).

	Allelic distribution																
	(retrospective study on *M. tuberculosis* isolates, *n* = 919)																Prospective study from fresh samples (*n* = 79)
	Allele (frequency, %)																
LOCUS	0	1	1s^a^	2	2s^a^	3	3s^a^	4	5	6	7	8	9	10	11	12	Missing allele (%)
**4156**	0.7	2.8		50.3		45.8		0.3									48.1
**1644**		10.3		10.2		68.9		10.4	0.1								44.3
**960**		0.1		4.9		46.0		13.0	33.7	1.3	0.6	0.1		0.1	0.1		38
**3192**		0.3		11.6		82.0		5.1	0.9	0.1							38
**2163b**		0.9		14.0		23.4		31.0	19.1	9.5	1.5	0.3		0.1	0.1		33
**4052**	0.1	0.4		4.7		12.2		14.4	24.1	23.3	13.2	3.0	2.7	0.5	0.6	0.6	30.3
**3690**		2.2		6.0		74.7		10.8	3.8	0.3	1.6	0.2	01	-	0.1	0.1	26.6
**4348**		1.3		97.7		1.0											25.3
**3007**		1.0		8.6		87.5		2.8									24
**3171**		0.2		7.6		88.6		2.7	0.6	0.1		0.1					22.8
**2401**		2.3		49.0		1.6		43.8	3.1								21.5
**2531**		0.3		0.1		4.5		2.2	80.5	12.0	0.4						21.5
**2059**		4.9		95.0		0.1											21.5
**2687**	0.2	99.2		0.5		0											21.5
**1955**		11.4		38.5		44.7		2.6	0.8	1.7		0.1	0.1				19
**2461**		1.7		95.0		2.6		0.4	0.2								19
**2165**		0.4		18.3		70.4		10.4	0.3		0.1						17.7
**2347**		0		5.9		0.7		91.5	1.8								17.7
**802**	0.1	4.1		24.6		36.9		23.1	7.8	2.8	0.5						16.4
**424**	0.1	4.3		73.2		15.0		7.1	0.2								16.4
**154**		5.0		91.6		3.4		0									16.4
**2996**		1.7		0.5		2.3		11.2	78.0	3.3	0.9	0.1					15.2
**577**		0.7		3.7		56.0		36.8	2.7	0.1							14
**580**	0.2	4.8	0.4	81.06	0.2	10.6	0.2	2.3	0.1								7.9

a Variant alleles specifically observed for locus 0580. These “s” alleles consist of *n*×77-bp repeat units (taken into account for allele coding), in contrast to classical alleles of *n*×77-bp repeat units (taken into account for allele coding), plus a single 53-bp repeat unit (not taken into account for allele coding).

## Discussion

To the best of our knowledge, this is the first prospective study to evaluate the possibility of *M. tuberculosis* genotyping directly from fresh clinical samples using the standardized 24-locus MIRU-VNTR typing method established for culture. The results clearly show that this typing can be performed directly on fresh clinical samples with high smear-positivity rates. The benefit in terms of timing is significant because the median time needed from specimen extraction to final genotyping result in this study was 1 day opposed to the 6 to 10 days needed to obtain genotyping results from cultured *M. tuberculosis*.

A previous study by Mokrousov et al. [15] used 12-locus MIRU-VNTR typing complemented by subtyping with three hypervariable loci directly on DNA extracted from sputum samples with a commercial kit, to characterize *M. tuberculosis* strains from HIV negative pulmonary TB patients from Kyrgyzstan. Complete genotypes were remarkably obtained for the 56 smear-positive samples tested. Although information on corresponding smear grades was not available, it could be speculated that bacterial loads were probably high in many of the cases, given the penitentiary population involved, often exposed elsewhere to delays in medical evaluation and treatment.

A very good potential for direct *M. tuberculosis* typing from early clinical samples was also shown in a study by Alonso et al., with complete genotypes obtained on all but one of 61 patient specimens[14]. This study was performed retrospectively on selected, stored samples in vast majority with high bacterial grades. Such samples were not representative of routine samples as we find them in our laboratory, in terms of varying quantity, smear quality (see below), and microscopic positivity. This study also included column-based purification of *M. tuberculosis* genomic DNA, which might also plausibly have contributed to the high rate of genotype completion as for the study mentioned above, as well as purification of PCR products, which is not part of the common routine workflow for typing cultured isolates.

Another study by De Beer et al. [21] evaluated the analytical sensitivity of VNTR typing version, by serial dilutions of DNAs from a few selected *M. tuberculosis* strains in samples from pooled bronchoalveolar lavage fluid (BALF), not reflecting the variable range of sample qualities seen in routine in every TB laboratory [21]. Despite these less harsh and artificial conditions using homogenized BALF samples, and the use of a so-called optimized version of VNTR typing, their results in terms of obtaining complete genotypes seemed to be much lower than in our prospective study using real conditions.

Our results show that successful 24-locus MIRU-VNTR typing directly from clinical samples is significantly associated with the higher bacterial load present in the sample. As expected, a higher correlation (*R* = 0.654) between smear positivity and completeness of MIRU-VNTR genotypes was observed for the patients' first 26 samples (*p* = 0.0003) compared to the 53 follow-up samples (R = 0.415, *p* = 0.002; Fig. 2). Many of these follow-up samples were collected weeks after the treatment onset, possibly resulting in lower mycobacterial DNA integrity. However, our results show that even follow-up samples with sufficient bacterial loads can be used for typing, which can be useful e.g. to evaluate potential laboratory cross-contamination or TB transmission events during the treatment follow-up phase.

This study suggests that the degree of typeability also depends on certain characteristics of clinical samples related to individual patients. We suspect that the high amount of mucus and saliva or inhibitors in samples from some patients negatively influenced efficient inhibitor-free DNA extraction. At least two cases have to be pointed out in this regard. Eleven samples from a first patient were collected (from day 1 after recognizing the patient as a new TB case, until day 83 after diagnosis) with different bacterial loads (3+, 1+, 2AFB, 1AFB). Only one sample collected at day 28, graded 1+, could not be fully typed (Table 1, patient KSA). In contrast, among the seven samples from another patient, taken from day 3 to 43 after diagnosis and with bacterial loads of 1+, 2AFB, 1AFB, and NEG, none could be fully typed, with a relatively high number of non-amplified loci (Table 1, patient HHA). These observations illustrate that studies where samples are preselected or simulated in conditions not reflecting conditions of diverse sputum samples can less predict the real potential and sensitivity of VNTR typing on clinical samples.

Complete typing results were obtained from 18 out of the 26 patients in total. In the remaining eight patients, a minimum of 16 loci could be amplified. To evaluate the potential significance of the alleles missing in the incomplete genotypes in terms of interpretation of molecular clustering in our epidemiological context, we analyzed the allelic distribution of the corresponding loci among 919 isolates from an ongoing 3-year, countrywide population-based study. This comparative analysis indicated a varying degree of importance, depending on the locus. Locus 4156, which was the most frequent single locus missing among clinical samples included in this study, shows a low allelic diversity in our strain population (Table 2). Therefore, there is a high likelihood that the molecular clustering defined on the basis of the remaining amplified loci would not change if 4156 were amplified. In contrast, a locus such as 2163b shows one of the highest levels of allelic diversity in our Slovenian strain population. Thus, when such a locus is missing, the strength of the molecular clustering based on the remaining loci is more uncertain and, hence, predictions about possible recent transmission are of lower confidence (Table 2) [11], [14]. Of course, the strength will proportionally decrease with an increasing number of missing alleles. Importantly, however, even incomplete genotyping results with a substantial number of missing loci can be exploited to quickly rule out suspected cross-contamination or TB transmission events as soon as other genotypes in the comparison differ by at least two loci amplified. This threshold of double-locus variation for reliable exclusion of a clonal link has been extensively calibrated and ensured in previous studies [22].

To introduce direct typing of *M. tuberculosis* from fresh clinical samples into prospective routine practice, we evaluated the associated cost/benefit ratio, taking into account the relatively lower typeability of low smear microscopy grade samples (Table S1). Under our conditions, the calculation indicates that direct 24-locus MIRU-VNTR typing would be cost-effective only in the case of highly positive samples, for which rapid typing results are needed to rule out or indicate the possibility of cross-contamination or TB transmission while contact tracing is still ongoing.

In conclusion, our prospective study demonstrated that *M. tuberculosis* genotyping using the 24-locus MIRU-VNTR method can be performed directly on fresh clinical samples using standard procedures originally developed for bacterial culture. Direct typing is more successful using early samples with high smear grades, although the nature and quality of the sample may influence the typeability. Our direct typing on clinical samples produced results that were as conclusive as those based on cultures for 20 out of 26 TB cases. Although it is probably possible to obtain a higher degree of genotype completion by including a column-based DNA purification step, the current protocol can be used even without such optimization to quickly resolve some urgent epidemiological questions and potential laboratory cross-contamination issues. As a result of this study, the policy for using mycobacterial typing has already partly changed in our country. We now perform direct typing on clinical samples in coordination with our epidemiologist, when urgent results are needed and the patient is highly contagious, i.e. with a smear positivity of 2+ or 3+, which incidentally also increases the chances of obtaining a complete strain genotype.

## Supporting Information

Table S1 **MIRU-VNTR typing results of** ***M*** **.** ***tuberculosis*** **from all first samples taken from newly detected TB patients (** ***n*** **= 26) correlated with smear microscopy result.**
(DOC)

Table S2 **MIRU-VNTR 24-locus typing results of 25 samples with the highest bacterial load in which the result of smear microscopy was 3+.** Missing alleles are coded as *na* (i.e., non amplified).
(DOC)
