# Supplementary material for: Prospective Genotyping of Mycobacterium tuberculosis from Fresh Clinical Samples
Source: PLoS One. 2014 Oct 14;9(10):e109547. doi: 10.1371/journal.pone.0109547 (PMC4196917; doi:10.1371/journal.pone.0109547)
Supplement: Table S1 — MIRU-VNTR typing results of M . tuberculosis from all first samples taken from newly detected TB patients ( n = 26) correlated with smear microscopy result. (DOC) [file pone.0109547.s001.doc]

**Table S1.** MIRU-VNTR typing results of *M*. *tuberculosis* from all first samples taken from newly detected TB patients (*n* = 26) correlated with smear microscopy result.

|  | Price (reagents) | 3+ | 2+ | 1+ | 2AFB | 1AFB | Σ |
| --- | --- | --- | --- | --- | --- | --- | --- |
| Typing successful first time | €54 | 5 | 2 |  |  |  | **7** |
| Typing successful in second trial | €108 | 6 | 1 | 2 |  |  | **9** |
| Typing not completely successful | €108 | 2 |  | 2 | 1 | 3 | **8** |
| No result | €108 |  |  | 1 | 1 |  | **2** |
| Σ |  | **13** | **3** | **5** | **2** | **3** | **26** |
