# Supplementary material for: Prospective Genotyping of Mycobacterium tuberculosis from Fresh Clinical Samples
Source: PLoS One. 2014 Oct 14;9(10):e109547. doi: 10.1371/journal.pone.0109547 (PMC4196917; doi:10.1371/journal.pone.0109547)
Supplement: Table S2 — MIRU-VNTR 24-locus typing results of 25 samples with the highest bacterial load in which the result of smear microscopy was 3+. Missing alleles are coded as na (i.e., non amplified). (DOC) [file pone.0109547.s002.doc]

**Table S2:** MIRU-VNTR 24-locus typing results of 25 samples with the highest bacterial load in which the result of smear microscopy was 3+. Missing alleles are coded as *na* (i.e., non amplified).

| **SAMPLE RECEIVED (D = DAY)** | **MICROSCOPY** | **580** | **2996** | **802** | **960** | **1644** | **3192** | **424** | **577** | **2165** | **2401** | **3690** | **4156** | **2163b** | **1955** | **4052** | **154** | **2531** | **4348** | **2059** | **2687** | **3007** | **2347** | **2461** | **3171** |
| --- | --- | --- | --- | --- | --- | --- | --- | --- | --- | --- | --- | --- | --- | --- | --- | --- | --- | --- | --- | --- | --- | --- | --- | --- | --- |
| 0D | 3+ | 2 | 4 | 2 | 3 | 3 | 3 | 2 | 4 | 3 | 2 | 1 | 2 | 3 | 2 | 5 | 2 | 6 | 2 | 2 | 1 | 3 | 4 | 2 | 3 |
| 1D | 3+ | 2 | 5 | 4 | 2 | 3 | 3 | 2 | 4 | 3 | 2 | 3 | 2 | 3 | 2 | 5 | 2 | 5 | 2 | 2 | 1 | 3 | 4 | 2 | 3 |
| 1D | 3+ | 2 | 5 | 2 | 4 | 3 | 3 | 2 | 3 | 3 | 4 | 4 | 3 | 4 | 3 | 6 | 2 | 5 | 2 | 2 | 1 | 3 | 4 | 2 | 3 |
| 3D | 3+ | 2 | 5 | 4 | 2 | 3 | 3 | 2 | 4 | 3 | 2 | 3 | 2 | 3 | 2 | 5 | 2 | 5 | 2 | 2 | 1 | 3 | 4 | 2 | 3 |
| 4D | 3+ | 2 | 5 | 2 | 4 | 3 | 3 | 2 | 3 | 3 | 4 | 4 | 3 | 4 | 3 | 6 | 2 | 5 | 2 | 2 | 1 | 3 | 4 | 2 | 3 |
| 4D | 3+ | 2 | 5 | 3 | 3 | 1 | 3 | 2 | 4 | 3 | 2 | 7 | 2 | 3 | 2 | 5 | 2 | 5 | 2 | 2 | 1 | 3 | 4 | 2 | 3 |
| 4D | 3+ | 2 | 5 | 3 | na | na | na | 2 | 3 | 3 | 4 | 3 | na | 3 | 3 | 5 | 2 | 5 | 2 | 2 | 1 | 3 | 4 | 2 | 3 |
| 5D | 3+ | 2 | 5 | 2 | 4 | 3 | 3 | 2 | 3 | 3 | 4 | 4 | 3 | 4 | 3 | 6 | 2 | 5 | 2 | 2 | 1 | 3 | 4 | 2 | 3 |
| 8D | 3+ | 2 | 5 | 4 | 3 | 1 | 3 | 2 | 4 | 2 | 2 | 5 | 2 | 2 | 2 | 5 | 2 | 5 | 2 | 2 | 1 | 3 | 4 | 3 | 2 |
| 8D | 3+ | 2 | 5 | 1 | 3 | 2 | 3 | 2 | 3 | 3 | 4 | 4 | 3 | 6 | 3 | 7 | 2 | 5 | 1 | 2 | 1 | 3 | 4 | 2 | 3 |
| 10D | 3+ | 2 | 5 | 4 | 2 | 3 | 3 | 2 | 4 | 3 | 2 | 3 | 2 | 3 | 2 | 5 | 2 | 5 | 2 | 2 | 1 | 3 | 4 | 2 | 3 |
| 11D | 3+ | 2 | 5 | 2 | 4 | 3 | 3 | 2 | 3 | 3 | 4 | 4 | 3 | 4 | 3 | 6 | 2 | 5 | 2 | 2 | 1 | 3 | na | na | na |
| 12D | 3+ | 2 | 5 | 3 | 3 | 3 | 3 | 2 | 3 | 3 | 4 | 3 | 3 | 3 | 3 | 5 | 2 | 5 | 2 | 2 | 1 | 3 | 4 | 2 | 3 |
| 13D | 3+ | 2 | 5 | 3 | 3 | 3 | 3 | 2 | 4 | 2 | 2 | 3 | 2 | 3 | 2 | 3 | 2 | 6 | 2 | 2 | 1 | 3 | 4 | 2 | 3 |
| 13D | 3+ | 2 | 5 | 2 | 5 | 3 | 3 | 2 | 3 | 3 | 4 | 3 | 3 | 4 | 3 | 6 | 2 | 5 | 2 | 2 | 1 | 3 | 4 | 2 | 3 |
| 14D | 3+ | 2 | 5 | 4 | 2 | 3 | 3 | 2 | 4 | 3 | 2 | 3 | 2 | 3 | 2 | 5 | 2 | 5 | 2 | 2 | 1 | 3 | 4 | 2 | 3 |
| 14D | 3+ | 2 | 5 | 3 | 3 | 3 | 3 | 2 | 3 | 3 | 4 | 3 | 3 | 3 | 3 | 5 | 2 | 5 | 2 | 2 | 1 | 3 | 4 | 2 | 3 |
| 15D | 3+ | 4 | 5 | 6 | 3 | 1 | 2 | 3 | 4 | 3 | 2 | 3 | na | 3 | 2 | 5 | 2 | 5 | 2 | 2 | 1 | 3 | 4 | 2 | 3 |
| 15D | 3+ | 2 | 5 | 3 | 5 | 3 | 3 | 2 | 3 | 3 | 4 | 3 | 3 | 4 | 3 | 4 | 2 | 5 | 2 | 2 | 1 | 3 | 4 | 2 | 3 |
| 19D | 3+ | 1s | 5 | 3 | 5 | 3 | 3 | 2 | 3 | 3 | 4 | 3 | 3 | 2 | 3 | 5 | 2 | 5 | 2 | 2 | 1 | 3 | 4 | 2 | 3 |
| 20D | 3+ | 2 | 5 | 2 | na | na | na | 2 | 3 | na | 4 | na | na | na | 3 | na | 2 | na | 2 | 2 | 1 | 3 | 4 | 2 | 3 |
| 21D | 3+ | 2 | 5 | 4 | 2 | 3 | 3 | 2 | 4 | 3 | 2 | 3 | 2 | 3 | 2 | 5 | 2 | 5 | 1 | 2 | 1 | 3 | 4 | 2 | 3 |
| 24D | 3+ | 2 | 5 | 4 | 2 | 3 | 3 | 2 | 4 | 3 | 2 | 3 | 2 | 3 | 2 | 5 | 2 | 5 | 2 | 2 | 1 | 3 | 4 | 2 | 3 |
| 25D | 3+ | 2 | 5 | 2 | 4 | 3 | 3 | 2 | 3 | 3 | 4 | 4 | 3 | 4 | 3 | 6 | 2 | 5 | 2 | 2 | 1 | 3 | 4 | 2 | 3 |
| 36D | 3+ | na | na | na | na | na | na | na | na | na | na | na | na | na | na | na | na | na | na | na | na | na | na | na | na |
